# Generative Adversarial Training for Supervised and Semi-supervised Learning

**DOI:** 10.3389/fnbot.2022.859610

**Published:** 2022-03-24

**Authors:** Xianmin Wang, Jing Li, Qi Liu, Wenpeng Zhao, Zuoyong Li, Wenhao Wang

**Affiliations:** ^1^Institute of Artificial Intelligence and Blockchain, Guangzhou University, Guangzhou, China; ^2^Fujian Provincial Key Laboratory of Information Processing and Intelligent Control, Minjiang University, Fuzhou, China; ^3^State Key Laboratory of Information Security, Institute of Information Engineering, Chinese Academy of Sciences, Beijing, China

**Keywords:** neural networks, adversarial training, generative AT, worst-case perturbations, smoothness function, trajectory-preserving-based alternating update strategy

## Abstract

Neural networks have played critical roles in many research fields. The recently proposed adversarial training (AT) can improve the generalization ability of neural networks by adding intentional perturbations in the training process, but sometimes still fail to generate worst-case perturbations, thus resulting in limited improvement. Instead of designing a specific smoothness function and seeking an approximate solution used in existing AT methods, we propose a new training methodology, named Generative AT (GAT) in this article, for supervised and semi-supervised learning. The key idea of GAT is to formulate the learning task as a minimax game, in which the perturbation generator aims to yield the worst-case perturbations that maximize the deviation of output distribution, while the target classifier is to minimize the impact of this perturbation and prediction error. To solve this minimax optimization problem, a new adversarial loss function is constructed based on the cross-entropy measure. As a result, the smoothness and confidence of the model are both greatly improved. Moreover, we develop a trajectory-preserving-based alternating update strategy to enable the stable training of GAT. Numerous experiments conducted on benchmark datasets clearly demonstrate that the proposed GAT significantly outperforms the state-of-the-art AT methods in terms of supervised and semi-supervised learning tasks, especially when the number of labeled examples is rather small in semi-supervised learning.

## 1. Introduction

Neural networks have launched a profound reformation in various fields, such as intelligent driving (Feng et al., 2021), neuro-inspired computing (Zhang et al., 2020; Deng et al., 2021b), smart health (Khan et al., 2021), and human computer interaction (Deng et al., 2021a; Pustejovsky and Krishnaswamy, 2021; Fang et al., 2022). However, in practical classification and regression applications (Wu et al., 2021a), since the number of training examples is finite, the error rate calculated by the training examples may be considerably deviated from the one by test examples. This fact causes the overfitting problem (Wu et al., 2021b), which greatly impacts the generalization performance of neural networks. In order to prevent the neural networks from overfitting, one popular approach is to augment the loss function by introducing a regularization term, which encourages the model to be less dependent on the empirical risk for the finite training examples. Based on Bayesian theory, this regularization term can be interpreted as a prior distribution reflecting the preconceived notion of the model (Bishop and Nasser, 2006; Wu et al., 2020). Accordingly, the prior distribution of a model is usually assumed to be smooth. That is to say, the outputs of a naturally occurring system tend to be smooth with respect to the spatial or temporal inputs (Wahba, 1990). This assumption indicates that the data points close to each other should be highly likely to infer the same predictions. Unfortunately, recent studies show that most of the neural networks suffer from misclassifying some data points that have only small differences from the correctly classified data points (Goodfellow et al., 2014b; Strauss et al., 2017; Yuan et al., 2019). These misclassified data points are called the adversarial examples, which are crafted by the addition of some imperceptive perturbations to the natural examples in the input space.

To overcome the problem that the neural networks are vulnerable to small but malicious perturbations, adversarial training (AT) is proposed (Goodfellow et al., 2014b; Wang et al., 2019; Cui et al., 2021; Zhang et al., 2022). AT aims to smooth the model outputs by penalizing the deviations caused by the adversarial perturbations. The major challenge of AT is how to accurately estimate such perturbations that alter the output distribution around the input data points. To this end, several perturbation-based methods have been proposed by solving an internal optimization problem at the current status of the model. For instance, random AT (RAT) (Zheng et al., 2016) improves the model smoothness by adding the randomly generated perturbations to the input data. These perturbed data points are encouraged to produce the same prediction given by its corresponding unperturbed versions. Since the perturbations around the input appear in random directions, RAT is referred to as an isotropic smoothing approach. However, it is shown that the isotropic smoothing makes the model particularly sensitive to adversarial examples (Szegedy et al., 2013; Goodfellow et al., 2014b). Based on this consideration, Goodfellow et al. (2014b) proposed a standard AT (SAT). SAT is an anisotropic method that smoothes the output distribution by making the model robust against perturbations in a specific direction. This specific direction in the input space is called the adversarial direction, in which the output of the model is the most sensitive. To identify the perturbations in the adversarial direction, SAT first formulates an objective function based on the differences between the prediction and correct labels and then solves this function with an efficient Frank-Wolfe optimizer. SAT requires the use of labels when calculating the adversarial perturbations. Hence, SAT cannot be applied to the regime of semi-supervised learning. Virtual AT (VAT) (Miyato et al., 2018) extends the notion of SAT in the sense that it defines the adversarial direction without label information, and thus can be applied to both supervised and semi-supervised learning tasks. We observe that in order to generate the adversarial perturbations, the existing AT methods explicitly define a smoothness function to regularize the neural networks. This leads to two limitations. First, it is extremely difficult to find a universal smoothness function due to the various output patterns and distance metrics. Second, there is no analytical solution to such a box-constrained function. Consequently, a numerical method is generally used to seek an approximate solution, which greatly affects the performance of identifying the worst-case adversarial perturbations.

Different from previous methodologies, we propose a novel AT methodology, named generative AT (GAT) in this article, to improve the smoothness of output distribution of neural networks for the supervised and semi-supervised learning tasks. The objective of the proposed GAT is to train the target classifier such that it not only achieve the minimum prediction error but also has the best robustness against the adversarial perturbations. To this aim, we formalize the regularizing process as a minimax game. To be specific, we exploit the cross entropy method to construct a new *adversarial loss* function. Moreover, we develop an effective alternating update strategy to optimize the challenging non-convex problems. The experimental results tested on benchmark datasets show that the proposed GAT obtains the empirical equilibrium point and state-of-the-art performance.

The main contributions of this article are summarized as follows:

- We formulate the regularizing for the learning task as a minimax game according to the outputs of the target classifier from the natural example and its adversarial version derived by a perturbation generator. As the game approaches the empirical equilibrium, the target classifier achieves the best performance.
- A new *adversarial loss* function is constructed based on the *cross entropy* method, which not only accurately reflects the deviation caused by the perturbation but also efficiently assesses the confidence of network output.
- An effective alternating update strategy based on trajectory preserving is proposed to control the minimax optimization training to be stable.
- The proposed GAT regularizes the model without label information, hence it can be applied to the supervised and semi-supervised learning tasks.

It is worth emphasizing that our method differs from any one of the generative-model-based AT methods (Kingma et al., 2014; Maaløe et al., 2016; Salimans et al., 2016; Dai et al., 2017). This family of methods is considered to be an improvement of Generative Adversarial Network (GAN), in the sense that the target classifier in their frameworks is the extension of the GAN's discriminator serving for distinguishing the natural and generated examples. For our method, the discriminator is not the target classifier; instead, it is manually designed according to the outputs of the target classifier over the natural example and its adversarial version.

## 2. Problem Setting and Related Works

Without loss of generality, we consider the classification tasks in a semi-supervised setting. Let $x\in X=R^{I}$ be the input vector with *I*-dimension and $y\in Y=Z^{K}$ be the one-hot vector of labels with *K* categories. $D^{l}=\left\{x_{\left(i\right)}^{l},y_{\left(i\right)}^{l}|i=1,...,N^{l}\right\}$ and $D^{ul}=\left\{x_{\left(j\right)}^{ul}|j=1,...,N^{ul}\right\}$ denote the labeled and unlabeled dataset, where *N*^*l*^ and *N*^*ul*^ are the number of labeled and unlabeled examples. AT regularizes the neural network such that both the natural and perturbed examples output the intended predictions. That is, we aim to learn a mapping 𝔽:*X* → [0, 1]^*K*^ parameterized with θ ∈ Θ *via* solving the following optimization problem


(1) $$\begin{array}{l} \min\left\{L_{S}\left(D^{l},\theta \right)+\lambda \cdot L_{R}\left(D^{l},D^{ul},\theta \right)\right\}. \end{array}$$

The symbol $L_{S}$ in Equation 1 represents the *supervised loss* over the labeled dataset, which can be expanded as


(2) $$\begin{array}{l} L_{S}=E_{\left(x^{l},y^{l}\right)\sim D^{l}}\Gamma \left(y^{l},{\text{F}}_{\theta }\left(x^{l}\right)\right), \end{array}$$

where ${\text{F}}_{\theta }\left(x^{l}\right)$ denotes the output distribution vector of the neural network on the input *x*^*l*^ given the model parameter θ, *y*^*l*^ is the one-hot vector of the true label for *x*^*l*^. The operator Γ(·, ·) denotes the distance measure used to evaluate the similarity of two distributions. A common choice of Γ for the supervised cost $L_{S}$ is the measure of *cross entropy*. $L_{R}$ is the *adversarial loss*, which is served as a regularization term for promoting the smoothness of the model. The *adversarial loss* plays an important role in enhancing the generalization performance while the number of labeled examples is small relative to the number of the whole training examples (i.e., *N*^*l*^ < < *N*^*ul*^+*N*^*l*^). λ is a non-negative value that controls the relative balance between the *supervised loss* and the *adversarial loss*.

Many approaches are presented to construct $L_{R}$ based on the smoothness assumption, which can be generally represented in a framework as


(3) $$\begin{array}{l} L_{R}={\text{E}}_{x\sim D}\Gamma \left({\text{F}}_{\theta }\left(x;\xi \right),{\tilde{\text{F}}}_{\theta^{\prime }}\left(x;\xi^{\prime }\right)\right), \end{array}$$

where *x* is sampled from the dataset D which consists of both labeled and unlabel examples. $\Gamma \left({\text{F}}_{\theta }\left(x;\xi \right),{\tilde{\text{F}}}_{\theta^{\prime }}\left(x;\xi^{\prime }\right)\right)$ is termed as the smoothness function, which is comprised of a teacher model **F**_θ_(*x*; ξ) and a student model ${\tilde{\text{F}}}_{\theta^{\prime }}\left(x;\xi^{\prime }\right)$. The teacher model is parameterized with parameter θ and perturbation ξ, while the student model is parameterized with parameter θ′ and perturbation ξ′. The goal of $L_{R}$ is to improve the model's smoothness by forcing the student model to follow the teacher model. That is to say, the output distributions yielded by $\tilde{\text{F}}$ is supported to be consistent with the outputs derived by **F**. To this end, the teacher model, student model, and similarity measure are required to be carefully crafted for formulating an appropriate smoothness function against the perturbation of the input and the variance of the parameters. Based on the implementations of this smoothness function, some typical AT approaches can be explicitly defined.

**Random Adversarial Training:** In RAT, random noises are introduced in the student model instead of the teacher model, and the parameters of the student model are shared with the teacher model. Moreover, *L*_2_ distance is used to measure the similarity of the output distributions derived by $\tilde{\text{F}}$ and **F** on the whole training examples. That is, θ′ = θ, $\xi^{\prime }\sim N\left(0,1\right)$, ξ = 0, and $D=D^{ul}⋃D^{l}$ for Equation 3.

**Adversarial Training With** **Π-Model:** In contrast to RAT, Π-model introduces random noises to both the teacher model and student model, i.e., $\xi^{\prime },\xi \sim N\left(0,1\right)$. The reason for this is based on the assumption that predictions yielded by natural example may itself be an outlier, hence it is reasonable to make two noisy predictions learn from each other. In this case, optimizing the smoothness function for Π-model is equivalent to minimizing the prediction variance of the classifier (Luo et al., 2018).

**Standard Adversarial Training:** Instead of adding random noises to the teacher/student model, the perturbation adopted in SAT is some imperceptible noise that is carefully designed to fool the neural network. The *adversarial loss* $L_{R}^{sat}$ of SAT can be written as


(4) $$\begin{array}{l} \begin{array}{l} L_{R}^{sat}={\text{E}}_{\left(x^{l},y^{l}\right)\sim D^{l}}\text{KL}\left(y^{l}||{\tilde{\text{F}}}_{\theta }\left(x^{l};\xi_{adv}\right)\right)\\ s.t.\;\xi_{adv}=\underset{\xi ;||\xi ||\leq \varepsilon }{\arg\max}\text{KL}\left(y^{l}||{\tilde{\text{F}}}_{\theta }\left(x^{l};\xi \right)\right), \end{array} \end{array}$$

where the operator KL(·||·) denotes the similarity measure of *Kullback-Leibler (K-L) divergence*. ξ_*adv*_ denotes adversarial perturbation which is added into *x*^*l*^ to make the output distribution of the student model most greatly deviate *y*^*l*^. ε is a prior constant that controls the perturbation strength. Note that the teacher model, in this case, is degenerated into the one-hot vector of the true label. Generally, we cannot obtain the exact adversarial direction of ξ_*adv*_ in a closed form. Hence, a linear approximation of this objective function is applied to approximate the adversarial perturbation. For ℓ_∞_ norm, the adversarial perturbation ξ_*adv*_ can be efficiently approximated by using the famous fast gradient sign method (FGSM) (Madry et al., 2017). That is,


(5) $$\begin{array}{l} \xi_{adv}\approx \varepsilon \cdot \text{sign}\left(\nabla_{x^{l}}\text{KL}\left(y^{l}||{\tilde{\text{F}}}_{\theta }\left(x^{l};\xi \right)\right)\right). \end{array}$$

Some alternative invariants such as the iterative gradient sign method (IGSM) (Tramèr et al., 2017) and the momentum IGSM (M-IGSM) (Dong et al., 2018) are available to solve the objective function. By adding adversarial perturbations to the student model, SAT obtains better generalization performance than RAT and Π-model. Unfortunately, SAT can only be applied in supervised learning tasks since it has to use the labeled examples to compute the *adversarial loss*.

**Virtual Adversarial Training:** Different from SAT, the key idea of VAT is to define the *adversarial loss* based on the output distribution inferred on the unlabeled examples. In this regard, the *adversarial loss* $L_{R}^{vat}$ of VAT can be written as


(6) $$\begin{array}{l} \begin{array}{l} L_{R}^{vat}={\text{E}}_{x\sim D^{l}\cup D^{ul}}\text{KL}\left({\text{F}}_{\theta }\left(x\right)||{\tilde{\text{F}}}_{\theta }\left(x;\xi_{adv}\right)\right)\\ s.t.\;\xi_{adv}=\underset{\xi ;||\xi ||\leq \varepsilon }{\arg\max}\text{KL}\left({\text{F}}_{\theta }\left(x\right)||{\tilde{\text{F}}}_{\theta }\left(x;\xi \right)\right). \end{array} \end{array}$$

To obtain the adversarial perturbation ε_*adv*_, Miyato et al. (2018) proposed to approximate the objective function with a second-order Taylor's expansion at ε = 0. That is,


(7) $$\begin{array}{l} \xi_{adv}\approx \underset{\xi ;||\xi ||\leq \varepsilon }{\arg\max}\frac{1}{2}\xi^{T}H\left(x,\theta \right)\xi , \end{array}$$

where *H* is a Hessian matrix which is defined by $H\left(x,\theta \right)=\nabla \nabla_{\xi }\text{KL}\left({\text{F}}_{\theta }\left(x\right)||{\tilde{\text{F}}}_{\theta }\left(x;\xi \right)\right)$. This binomial optimization is an eigenvalue problem that can be solved using power iteration algorithm. Since VAT acquires the adversarial perturbation in the absence of label information, this method is applicable to both supervised and semi-supervised learning.

## 3. The Proposed Method

Adversarial training methods regularize the neural network *via* forcing the output distribution to be robust against adversarial examples. To obtain intentional perturbations, the existing AT methods require to explicitly define a smoothness function to compute the perturbations. Due to the non-convex characteristic of the smoothness function, the existing AT methods usually fail to generate worst-case perturbation by approximation analysis. To tackle this problem, we propose a novel AT framework termed GAT for improving the smoothness of the neural network, where the worst-case perturbation of the input is generated by a generator. In the following sections, we construct our framework by answering two central questions: (1) how to formulate the loss function with the perturbation generator and target classifier and (2) how to effectively optimize this loss function during the training process.

### 3.1. GAT Loss Based on Minimax Game

In our framework, two neural networks are considered, i.e., the target classifier **T**_θ_(*x*) parameterized with θ and the perturbation generator **G**_φ_(*x*) parameterized with φ. In our framework, the target classifier is the optimization objective that will be required eventually. The perturbation generator is constructed by an auto-encoder-like neural network. Specifically, the perturbation generator can be defined as a mapping **G**_φ_:X→X, which takes a natural example in X and then transforms it into an imperceptible perturbation in the same space X. For ℓ_∞_ norm, such constraints can be represented as


(8) $$\begin{array}{l} \forall x,||{\text{G}}_{\varphi }\left(x\right){||}_{\infty }\leq \varepsilon , \end{array}$$

where ε is the perturbation bounds that controls the adversarial strength. To implement the constraints indicated by Equation 8, the activation function of the last layer in **G**_φ_ is particularly defined as ε·*tanh*(·). Then, the generated perturbation is added into the corresponding natural example to composite an adversarial example.

The goal of **G**_φ_ is to find a perturbation that most deviates the current inferred output of the target classifier from the status quo, while **T**_θ_(*x*) is to minimize the prediction error for the natural example as well as the deviation caused by such perturbation. This problem can be formulated as a minimax game and the loss function of which can be formulated as


(9) $$\begin{array}{l} \underset{\theta }{\min}\underset{\varphi }{\max}\;E_{\left(x^{l},y^{l}\right)\sim D^{l}}\Gamma_{S}\left(y^{l},{\text{T}}_{\theta }\left(x^{l}\right)\right)\\ \;+\lambda \cdot E_{x\sim D^{l}\cup D^{ul}}\Gamma_{R}\left({\text{T}}_{\theta }\left(x\right),{\text{T}}_{\theta }\left({\text{G}}_{\varphi }\left(x\right)+x\right)\right). \end{array}$$

Equation 9 is referred to as the GAT loss, which is comprised of a *supervised loss* $L_{S}$ and an *adversarial loss* $L_{R}$ . $L_{S}$ is determined by labeled examples, while $L_{R}$ is independent of the labels and served as a regularization term smoothing the model. The parameter λ controls the balance of $L_{S}$ and $L_{R}$. For the maximization and minimization loop of the minimax game, φ and θ are the parameters required to be optimized. Since $L_{R}$ is defined over the whole data set, our method is applicable to semi-supervised learning. Note that for the *adversarial loss*, the target classifier **T**_θ_(*x*) is considered as the teacher model, while the compound function of **T**_θ_(**G**_φ_(*x*)+*x*) is served as the student model.

In addition, the operator Γ_*S*_(·, ·) and Γ_*R*_(·, ·) are the similarity measures for $L_{S}$ and $L_{R}$, respectively. Here, Γ_*R*_ is crucial for the construction of *adversarial loss*. Instead of using *K-L divergence* to define the *adversarial loss* as VAT/SAT does, we exploit *cross entropy* measures to formulate the *adversarial loss* function. There are two beneficial effects for this implementation. First, *cross entropy* overcomes the problem of zero avoiding, an inward nature for the *K-L divergence*(Bishop and Nasser, 2006). Second, since *cross entropy* can be represented as the sum of *K-L divergence* and *information entropy*, $L_{R}$ not only implies the deviation of the output distributions, but also signifies the confidence of the prediction of the target classifier. In particular, by substituting Γ_*R*_ with *cross entropy* in Equation 9, $L_{R}$ in GAT loss can be rewritten as


(10) $$\begin{array}{l} \begin{array}{l} \text{CE}\left({\text{T}}_{\theta }\left(x\right),{\text{T}}_{\theta }\left({\text{G}}_{\varphi }\left(x\right)+x\right)\right)\\ \;=\text{KL}\left({\text{T}}_{\theta }\left(x\right)||{\text{T}}_{\theta }\left({\text{G}}_{\varphi }\left(x\right)+x\right)\right)+\text{H}\left({\text{T}}_{\theta }\left(x\right)\right), \end{array} \end{array}$$

where the operator CE(·, ·) and H(·) denote *cross entropy* and *information entropy*. In Equation 10, KL(**T**_θ_(*x*)||**T**_θ_(**G**_φ_(*x*)+*x*)) is termed as smoothness term, which reflects the deviation of the output distributions, while H(**T**_θ_(*x*)) is termed as confidence term, which indicates the confidence of the output distribution. Moreover, we observed that the confidence term is independent with parameter φ. Hence, for the maximization loop of the minimax game, maximizing $L_{R}$ requires to maximize the smoothness term only. Whereas, for the minimization loop, minimizing $L_{R}$ requires to minimize both the smoothness term and confidence term. Note that minimizing the confidence term facilitates boosting of the prediction confidence of the neural network. Thus, our *adversarial loss* has the effect of entropy minimization proposed in Grandvalet and Bengio (2004) and Sajjadi et al. (2016).

### 3.2. Alternating Update Process Based on Trajectory Preserving

**Figure 1:**
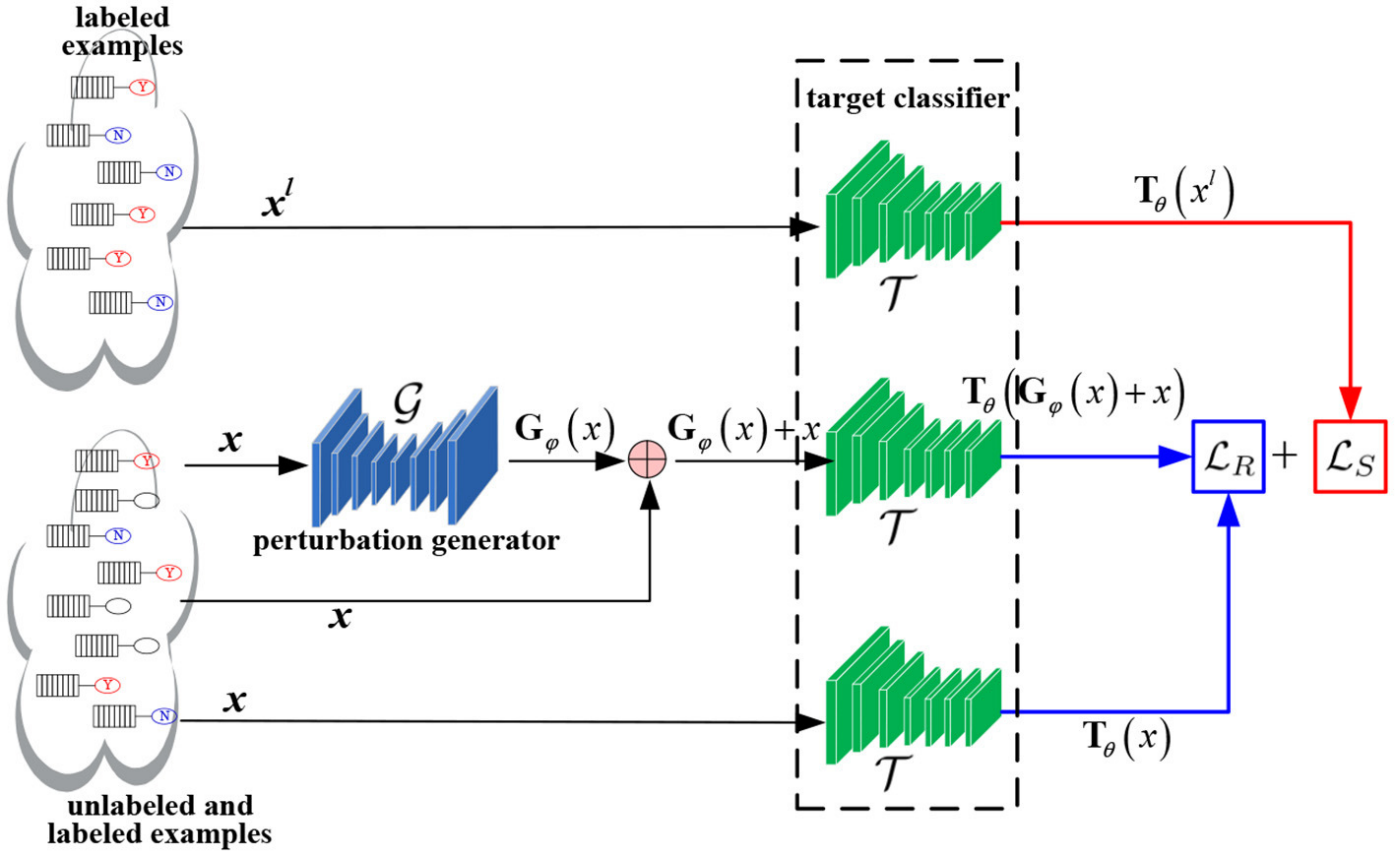
The overall framework of Generative AT (GAT).

Figure 1 depicts the framework of GAT, in which two neural networks are required to be optimized, i.e., the target classifier T and the perturbation generator G. G takes natural example *x* from the full dataset comprising of both the labeled and unlabeled examples and generates a perturbation **G**_φ_(*x*). Then, **G**_φ_(*x*) is appended into *x* to composite an adversarial example. Both the adversarial example and its corresponding natural example are fed into T for constructing the *adversarial loss* $L_{R}$. Meanwhile, labeled example *x*^*l*^ sampled from the labeled dataset is input to T for formulating the *supervised loss* $L_{S}$.

The objective of our framework is to find stable θ and φ such that G maximizes the GAT loss for the given fixed θ, while T minimizes the GAT loss for the given fixed φ. Due to the non-linear constraint of the perturbation and non-convex properties of the loss function, this optimization problem is very challenging. Inspired by the training pattern of GAN (Goodfellow et al., 2014a) and some common tricks in reinforcement learning (Mnih et al., 2015), we propose to optimize the GAT loss by an alternative updating procedure and stabilize this procedure based on trajectory preserving.

First, we decompose the minimax optimization problem into the inner loop and outer loop. The inner loop aims to derive an optimal φ for maximizing the loss, while the outer loop aims to obtain an optimal θ for minimizing the loss. Due to the fact that the parameter φ in the inner loop is independent of the *supervised loss* during the maximizing procedure, then the optimal φ of G under the fixed θ can be written as Equation 11. Meanwhile, the optimal θ of T under the given fixed φ can be represented as Equation 12.


(11) φ=arg max φEx~Dl∪DulCE(Tθ(x),Tθ(x+Gφ(x))),

(12) $$\begin{array}{l} \theta =\text{arg}\underset{\theta }{\min}\;E_{\left(x^{l},y^{l}\right)\sim D^{l}}\text{CE}\left(y^{l},{\text{T}}_{\theta }\left(x^{l}\right)\right)+\\ \;\lambda \cdot E_{x\sim D^{l}\cup D^{ul}}\text{CE}\left({\text{T}}_{\theta }\left(x\right),{\text{T}}_{\theta }\left(x+{\text{G}}_{\varphi }\left(x\right)\right)\right). \end{array}$$

Second, since the perturbation generator and the target classifier are assumed to be neural networks, the parameters θ and φ in Equations 11 and 12 can be calculated by stochastic-gradient-based methods (Liu et al., 2021; Jin et al., 2022). A traditional solution to this minimax problem is to alternatively update φ by gradient ascent over the full dataset and update θ by gradient descent over the labeled dataset. However, since the number of labeled training examples is small, both φ and θ are not easy to converge in practice. We develop a trajectory preserving strategy to tackle this problem. In our method, for each epoch of alternating, we update φ using gradient ascent and record the update trajectories of φ. Then, based on these trajectories, we retrieve the intermediate parameter φ′ by executing a pseudo-update procedure for φ. Finally, we update θ by gradient descent under the given φ′.

**Algorithm 1:**
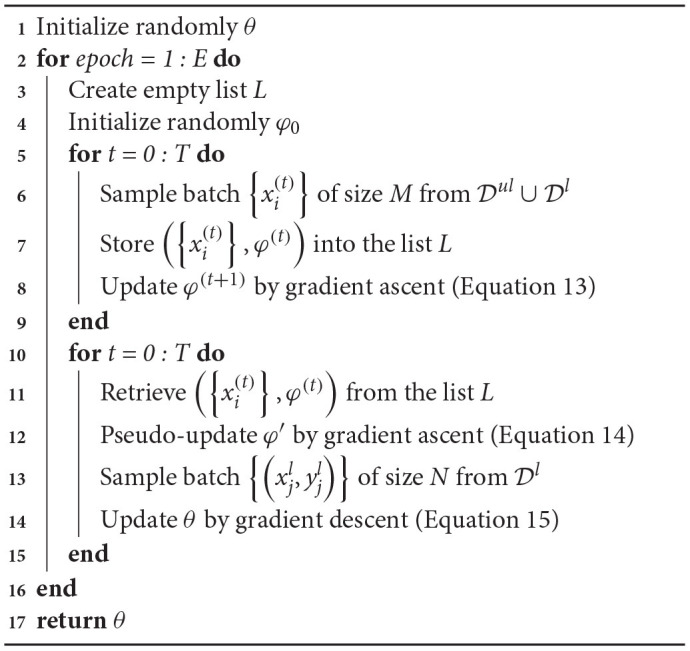
Trajectory preserving training process.

The implementation details of the proposed trajectory preserving training procedure are illustrated in Algorithm 1, where *E* is the number of training epochs, *T* is the maximum iterations in each epochs. Equations 13 and 14 represent the updating and pseudo-updating for φ by gradient ascent. Equation 15 describes the updating process for θ by gradient descent. α_*g*_ and α_*t*_ are the learning rate for the perturbation generator and target classifier, respectively.


(13) $$\begin{array}{l} \varphi^{\left(t+1\right)}=\varphi^{\left(t\right)}+\alpha_{g}\nabla_{\varphi^{\left(t\right)}}\frac{1}{M}\sum_{i=1}^{M}\text{CE}({\text{T}}_{\theta }\left(x_{i}^{\left(t\right)}\right),{\text{T}}_{\theta }\left(x_{i}^{\left(t\right)}+{\text{G}}_{\varphi^{\left(t\right)}}\left(x_{i}^{\left(t\right)}\right)\right)) \end{array}$$

(14) $$\begin{array}{l} \varphi^{\prime }=\varphi^{\left(t\right)}+\alpha_{g}\nabla_{\varphi^{\left(t\right)}}\frac{1}{M}\sum_{i=1}^{M}\text{CE}({\text{T}}_{\theta }\left(x_{i}^{\left(t\right)}\right),{\text{T}}_{\theta }\left(x_{i}^{\left(t\right)}+{\text{G}}_{\varphi^{\left(t\right)}}\left(x_{i}^{\left(t\right)}\right)\right)) \end{array}$$

(15) $$\begin{array}{l} \theta =\theta -\alpha_{t}\nabla_{\theta }\{\frac{1}{N}\sum_{j=1}^{N}\text{CE}\left(y_{j}^{l},{\text{T}}_{\theta }\left(x_{j}^{l}\right)\right)+\frac{\lambda }{M}\sum_{i=1}^{M}\text{CE}({\text{T}}_{\theta }\left(x_{i}^{\left(t\right)}\right),\\ \;{\text{T}}_{\theta }\left(x_{i}^{\left(t\right)}+{\text{G}}_{\varphi^{\prime }}\left(x_{i}^{\left(t\right)}\right)\right))\}. \end{array}$$

## 4. Experiments

To validate the performance of our method on supervised and semi-supervised task, we carried out experiments on synthetic datasets and practical benchmarks by comparing with various strong competitors.

### 4.1. Supervised Learning on a Synthetic Dataset

This section tests the supervised learning performance of our method for binary classification problems using two well-known synthetic datasets, i.e., the “Moons” dataset (termed as *M*-dataset) and the “Circles” dataset (termed as *C*-dataset). The data points in the two datasets are sampled uniformly from two trajectories over the space of *R*^2^ and embedded linearly into 100-dimension vector space. Each dataset contains 16 training data points and 1,000 testing points. **Figures 4**, **5** provide the visualizations for *M*-dataset and *N*-dataset, where the red circles and blue triangles separately stand for the training examples with labels 1 and 0. The target classifier used in this experiment is a neural network with one hidden layer comprised of 100 hidden units, where ReLU and softmax activation function are applied to the hidden units and output units. We compare our method with some popular AT methods, such as SAT (Goodfellow et al., 2014b), RAT (Zheng et al., 2016), and VAT (Miyato et al., 2018). These AT methods and the proposed GAT are conducted under the setting of λ = 1 and ϵ = 0.2. Particularly, the perturbation generator in our method has three hidden layers with the unit number 128, 64, and 128, respectively.

**Figure 2:**
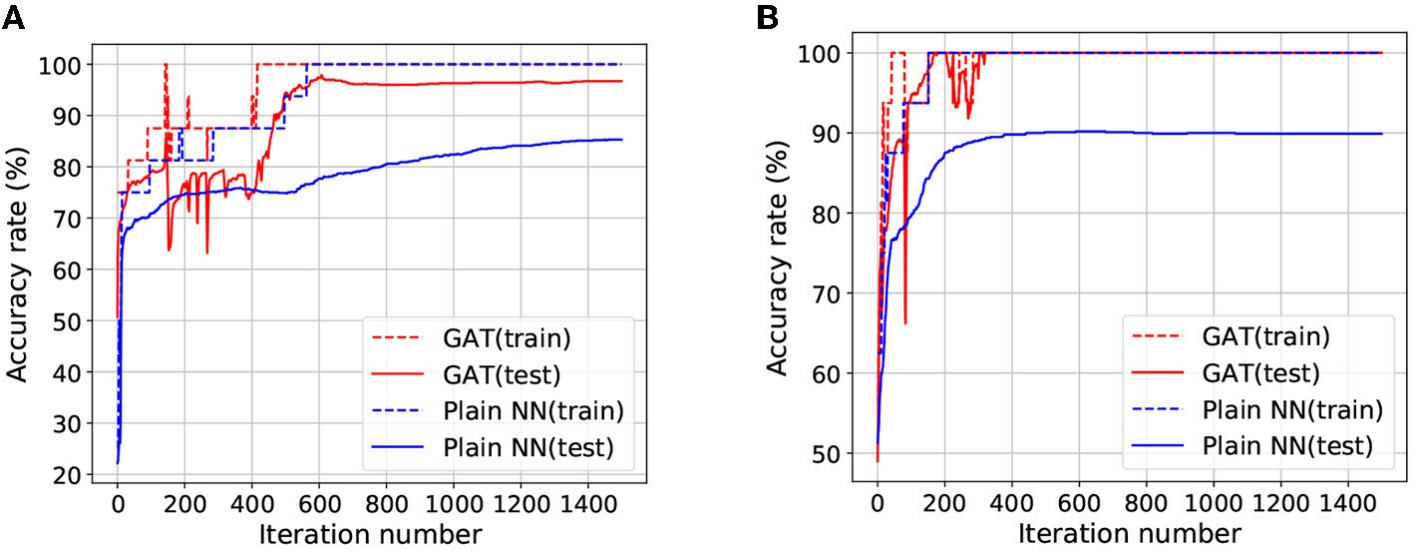
The transition curves of accuracy rates by Plain NN and the proposed GAT on *M*-dataset and *C*-dataset. **(A)** Plots the results for *M*-dataset, **(B)** plots the results for *C*-dataset.

**Figure 3:**
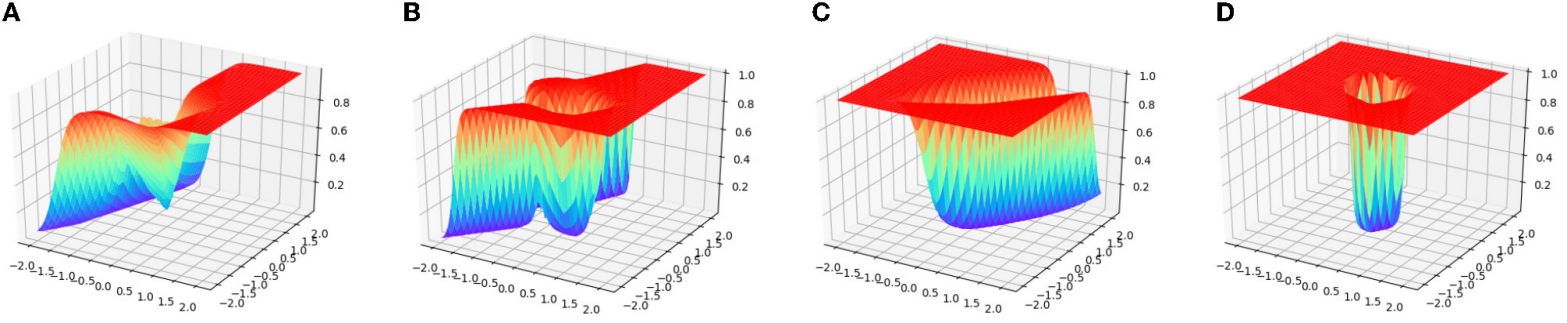
The visualization of model distributions of GAT and Plain NN on the synthetic datasets. **(A,B)** Show the distribution surface on *M*-dataset, **(C,D)** show the distribution surface on *C*-dataset, where flat surface regions implicate small output deviations.

Since the number of the training examples is extremely small compared to the input dimension, the target classifier for binary classification is very vulnerable to the problem of overfitting. Figures 2A,B depict the transitions of the accuracy rates for the target classifier with the GAT regularization and without this regularization (termed as Plain NN). It can be observed that the training accuracy of Plain NN and GAT achieved 100% for the two datasets. Nevertheless, the test accuracy rate of GAT is noticeably higher than that of Plain NN. Although our method suffers from some fluctuations with the accuracy rate at the initial stage of the training process, the test accuracy rate of our method finally achieves a stable value after a few iterations, thanks to the trajectory preserving training strategy. Figure 3 visualizes the output distributions of the trained target classifier on the *M*-dataset and *C*-dataset with our method and Plain NN. We can observe that compared to plain NN, GAT provides more flat regions for the landscape of the output distribution. This phenomenon indicates that our method is conducive to the smoothness of the model in the sense that flat surfaces of the landscape imply small deviations of the output.

**Figure 4:**
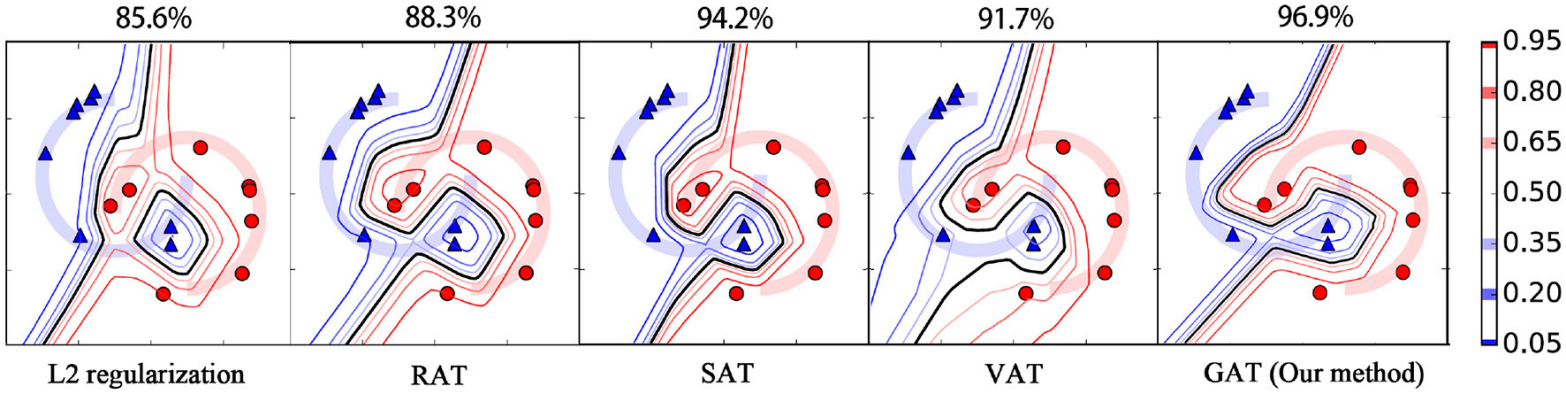
The contour of output confidences for label 1 on *M*-dataset with various regularization methods. The red circles and blue triangles represent the data points with labels 1 and 0, respectively. The decision boundaries with different confidences are plotted with different colored contours. Note that the black line represents the contour of probability value 0.5, which is usually served as the decision boundary for the binary classification task. The accuracy rate of each method for the test examples is displayed above the panel.

**Figure 5:**
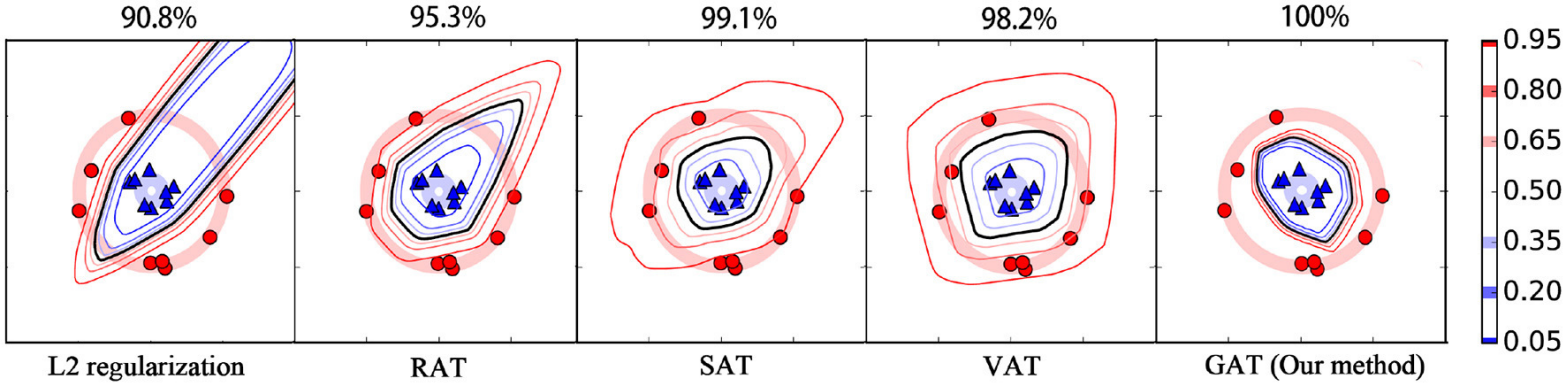
The contour of output confidence for label 1 on *C*-dataset with various regularization methods. The detailed illustrations for this figure can be referred to the caption of Figure 4.

Moreover, we plot the contours of the target classifier's predictions for label 1 on the two synthetic datasets by various regularization methods. As shown in Figures 4, 5, the black line in each plot stands for the contour of value 0.5, which is usually used as the decision boundary for the binary classification tasks. From these figures, we can see that the *L*_2_ regularization method fails to acquire correct decision boundary on both the *M*-dataset and *C*-dataset, hence, many false predictions are produced by this method. RAT obtains convincing decision boundary for *M*-dataset, but it generates an unreasonable decision boundary for *C*-dataset. Among these methods, only SAT, VAT, and our method yield applicable decision boundary for both the *M*-dataset and *C*-dataset, because these methods employ an anisotropic way to smooth the classifier. Compared to RAT and VAT, the decision boundaries of our method for different contour values are more compact. This phenomenon illustrates that our method can provide more confidence predictions for the new instances, thanks to the *cross entropy* measure for the adversarial loss. Our method also achieves the highest test accuracy rate against its competitors on both the *M*-dataset and *C*-dataset.

### 4.2. Supervised Learning on the Benchmark Dataset

In this section, we evaluate the performance of our methods on the MNIST dataset for a supervised learning scenario. The origin 60,000 training examples are split into 50,000 training examples and 10,000 test examples. The target classifier is made up of four hidden dense layers, whose unit numbers are 1200, 600, 300, and 150, respectively. The input dimension of the target classifier is 784 and the output dimension is 10. For each method, we use the setting of hyper-parameters that exhibits the best performance on the test dataset to train the neural network and record their test errors. The perturbation generator in our method is comprised of hidden layers whose unit numbers are 1200, 600, 300, and 600, respectively. The control parameters of the methods by our implementations are set λ = 1 and ϵ = 0.2. We compare our method with some typical AT methods on the MNIST dataset for supervised learning task. To verify the capability of the trajectory preserving strategy, we also conducted an ablation experiment for GAT-woTP, a method using the proposed GAT framework but Without Trajectory Preserving strategy during the training. The test error rates of these methods are reported in Table 1. The experimental results demonstrate that our method surpasses the previous state-of-the-art AT methods by a large margin. Moreover, our method also outperforms advanced generation-based algorithms such as Ladder network and CatGAN. Besides, note that the error rate obtained by our method is much lower than that acquired by GAT-woTP. This is because the trajectory preserving strategy is benefit to ensure the stability of the training process. Without this strategy, GAT is usually difficult to achieve a favorable convergent point during the training.

**Table 1:** Test error rates of various regularization methods for supervised learning task on MNIST dataset.

**Method**	**Test error rate (%)**
SVM (gaussian kernel)	1.40
Dropout	1.05
Maxout networks	0.94
DBM	0.79
Ladder network^†^	0.57
Conv-CatGAN^†^	0.48
Plain NN (Baseline)	1.15
RAT	0.85
SAT (*L*_∞_)	0.78
VAT	0.66
GAT-woTP	0.65
GAT (Our method)	0.45

*The upper panel refers to the experimental results reported in prior work, the error rates in the bottom panel are derived by our implementations. ^†^Represents the generation-based methods*.

### 4.3. Semi-supervised Learning on Benchmark Dataset

This section validates the effectiveness of our method for semi-supervised learning tasks on three popular benchmarks of MNIST, SVHN, and CIFAR-10. According to the experimental setups in Miyato et al. (2018), we take a test dataset with fixed size 1,000 from the training examples and train the classifier under four sizes of the labeled dataset, i.e., *N*_*l*_ = {100, 600, 1000, 3000}, where *N*_*l*_ is size of the dataset. The rest instances of the training examples are served as unlabeled examples. Then, we record the test errors under different values of *N*_*l*_. For our method, we use a mini-batch of size 64 to calculate the *supervised loss* in Equation 11 and a mini-batch of size 256 to calculate the *adversarial loss* in Equation 12. The control parameters of the methods by our implementations are set at λ = 1 and ϵ = 0.2. To test the performance of the trajectory preserving strategy for semi-supervised learning, we make several ablation experiments for GAT-woTP which is described in Section 4.2. For the reason that SAT can only be applied to supervised learning task, the results of SAT have not been reported in these experiments.

For the MNIST dataset, the structures of the target classifier and perturbation generator are identical to the structures employed in Section 4.2. Table 2 lists the test error rates of the comparing semi-supervised learning methods for different values of *N*_*l*_ on MNIST. The experimental results show that our method achieves the lowest error rates among all the methods for different numbers of labeled examples. Moreover, our method significantly outperforms the state-of-the-art AT methods when the number of labeled examples is small. For the experiments on SVHN and CIFAR-10, two type of convolution neural networks (CNNs), named “Small” (Salimans et al., 2016) and “Large” (Laine and Aila, 2018), are employed as the target classifiers. More details about the settings and structures of the two CNNs can be referred to (Miyato et al., 2018). The structure of the perturbation generator in this experiment is the same as the one applied in the experiment for the MNIST dataset. The performance of various comparing methods for SVHN and CIFAR-10 is reported in Table 3. From the table, we can find that GAT obtains the best generalization capability for the SVHN dataset and achieves comparable performance to the state-of-the-art generation-based method such as TNAR-VAE for the CIFAR-10 dataset. In addition, GAT reaches lower error rates compared to GAT-woTP for all the three benchmarks, which verifies the favorable performance of the trajectory preserving strategy for stabilizing the training for our proposal.

**Table 2:** Test error rates of semi-supervised learning methods on MNIST datasets.

**Method**	**Test error rate (%)**			
	***N*_*l*_ = 100**	***N*_*l*_ = 600**	***N*_*l*_ = 1, 000**	***N*_*l*_ = 3, 000**
SVM	23.44	8.85	7.77	4.21
EmbedNN	16.9	5.97	5.73	3.59
PEA	10.79	2.44	2.23	1.91
Conv-CatGAN^†^	1.93(±0.01)	1.86(±0.11)	1.73(±0.18)	1.67(±0.12)
Ladder networks^†^	1.06(±0.37)	0.93(±0.07)	0.84(±0.08)	0.79(±0.09)
Auxiliary DGM^†^	0.96(±0.02)	0.90(±0.05)	0.86(±0.13)	0.78(±0.05)
RAT	6.62(±1.02)	3.75(±0.14)	1.61(±0.09)	1.51(±0.08)
VAT	2.38(±0.11)	1.38(±0.08)	1.35(±0.12)	1.28(±0.07)
GAT-woTP	1.97(±0.87)	1.66(±0.85)	1.58(±0.96)	1.32(±0.65)
GAT (Our method)	0.90(±0.11)	0.85(±0.09)	0.83(±0.17)	0.75(±0.08)

*N_l_ denotes the number of labeled examples for the training dataset.*

*The results in the upper panel are referred to the reports in prior work, the error rates in the bottom panel are derived by our implementations. ^†^Represents the generation-based methods*.

**Table 3:** Test error rates (%) of semi-supervised learning methods on SVHN and CIFAR-10 datasets.

**Method**	**SVHN**	**CIFAR-10**
	***N*_*l*_ = 1, 000**	***N*_*l*_ = 4, 000**
Π-model	5.43(±0.25)	16.55(±0.29)
Mean teacher	5.21(±0.21)	17.74(±0.30)
ALI	7.41(±0.65)	17.99(±1.62)
Ban GAN^†^	4.25(±0.03)	14.41(±0.30)
Tripple GAN^†^	5.77(±0.17)	16.99(±0.36)
Improved GAN^†^	4.39(±1.20)	16.20(±1.60)
TNAR-LGAN (Small)^†^	4.25(±0.09)	12.97(±0.31)
TNAR-LGAN (Large)^†^	4.03(±0.13)	12.76(±0.04)
RAT (Small)	8.42(±0.22)	18.58(±0.26)
RAT (Large)	8.36(±0.22)	18.23(±0.16)
VAT (Small)	6.83(±0.24)	14.87(±0.13)
VAT (Large)	5.77(±0.32)	14.18(±0.38)
GAT-woTP (Small)	6.53(±0.95)	14.36(±1.03)
GAT-woTP (Large)	5.26(±0.92)	14.02(±0.88)
GAT (Our method, Small)	4.27(±0.14)	12.96(±0.15)
GAT (Our method, Large)	4.01(±0.11)	12.81(±0.13)

*N_l_ represents the number of labeled examples in the training dataset. The results in the upper panel are referred to the reports in prior work, the results in the bottom panel are derived from our implementations. ^†^Stands for the generation-based methods*.

## 5. Conclusion

In this article, a novel GAT framework has been proposed to improve the generalization performance of neural networks for both the supervised and semi-supervised learning tasks. In the proposed framework, the target classifier is regularized by letting the perturbation generator watch and move against the target classifier in a minimax game. We exploit the *cross entropy* to evaluate the output deviation for the regularization term such that the prediction of the target classifier can be reinforced. Furthermore, an effective alternating update method is developed to stably train the target classifier and perturbation generator. Numerous experiments are conducted on synthetic and real datasets and their results demonstrate the effectiveness of our proposal.

## Data Availability Statement

The raw data supporting the conclusions of this article will be made available by the authors, without undue reservation.

## Author Contributions

XW contributed to the conception of the study, performed the data analyses, and wrote the manuscript. JL contributed significantly to analysis and manuscript preparation. QL, WZ, ZL, and WW performed the experiments.

## Funding

This work was supported by the National Natural Science Foundation of China (Nos. 62072127 and 62002076), Project 6142111180404 supported by CNKLSTISS, Science and Technology Program of Guangzhou, China (Nos. 202002030131 and 201904010493), Guangdong Basic and Applied Basic Research Fund Joint Fund Youth Fund (No. 2019A1515110213), Open Fund Project of Fujian Provincial Key Laboratory of Information Processing and Intelligent Control (Minjiang University) (No. MJUKF-IPIC202101), Natural Science Foundation of Guangdong Province (No. 2020A1515010423).

## Conflict of Interest

The authors declare that the research was conducted in the absence of any commercial or financial relationships that could be construed as a potential conflict of interest.

## Publisher's Note

All claims expressed in this article are solely those of the authors and do not necessarily represent those of their affiliated organizations, or those of the publisher, the editors and the reviewers. Any product that may be evaluated in this article, or claim that may be made by its manufacturer, is not guaranteed or endorsed by the publisher.
